# Perioperative Complications and Oncologic Outcomes after Radical Cystectomy in End-Stage Renal Disease Patients with Bladder Cancer Obtained Using a Standardized Reporting System

**DOI:** 10.3390/cancers14143512

**Published:** 2022-07-19

**Authors:** Yu-Liang Liu, Chun-Te Wu, Yu-Chao Hsu, Miao-Fen Chen, Chih-Shou Chen, Chung-Sheng Shi, Yun-Ching Huang

**Affiliations:** 1 Division of Urology, Department of Surgery, Chang Gung Memorial Hospital at Chiayi, Chiayi 613016, Taiwan; armsliu@cgmh.org.tw (Y.-L.L.); cv7589@cgmh.org.tw (C.-S.C.); 2 Department of Medicine, College of Medicine, Chang Gung University, Taoyuan 333323, Taiwan; chuntewu@cgmh.org.tw (C.-T.W.); hsuyc@cgmh.org.tw (Y.-C.H.); miaofen@cgmh.org.tw (M.-F.C.); 3 Department of Urology, Chang Gung Memorial Hospital at Linkou, Taoyuan 333423, Taiwan; 4 Department of Radiation Oncology, Chang Gung Memorial Hospital at Chiayi, Chiayi 613016, Taiwan; 5 Graduate Institute of Clinical Medical Sciences, College of Medicine, Chang Gung University, Taoyuan 333323, Taiwan; csshi@mail.cgu.edu.tw

**Keywords:** complication, cystectomy, bladder, cancer, urothelial carcinoma, end-stage renal disease, dialysis

## Abstract

**Simple Summary:**

Urothelial carcinoma (UC) of the bladder is the most common malignancy in dialysis patients in Taiwan compared with renal cell carcinoma in Western countries. Radical cystectomy is the standard of care for localized muscle-invasive bladder cancer (MIBC), as well as for selected patients with high-risk non-MIBC. Despite improvements in anesthetic competency, surgical techniques, and postoperative care, dialysis patients undergoing radical cystectomy are at a higher risk of perioperative morbidity and mortality than non-dialysis patients. Due to the relative rarity of the condition, published data on predictors of outcome in ESRD patients undergoing radical cystectomy are scant. Our study revealed that accurate reporting of complications is necessary for preoperative counseling, identifying modifiable risk factors, and planning risk mitigation strategies. High comorbidity and low-volume surgeons were interrelated as notable risk factors for major complications. In addition to tumor-related factors, male sex, older age, and major complications significantly influence survival.

**Abstract:**

Background: We investigated the use of a standardized reporting system to study perioperative complications and oncologic outcomes after radical cystectomy in end-stage renal disease (ESRD) patients with bladder cancer. Methods: We reviewed retrospective outcomes in 141 ESRD patients with bladder cancer who underwent radical cystectomy between 2004 and 2015. Complications were graded using the Clavien–Dindo classification system with 0–2 classified as “No Major Complications” and Clavien 3–5 as “Major Complications”. Low-volume surgeons were classified as those performing fewer than nine cases during the study. Fisher’s exact test along with the chi-squared test, two-tailed *t* tests, logistic regression, and the Cox proportional hazard model were used to evaluate all clinically meaningful covariates. Results: Ninety-nine (99, 70.2%) patients had no major complications, and forty-two (29.8%) patients had major complications. Patients in the major complications group were older, had a higher Charlson comorbidity index (CCI), and had a longer hospitalization duration than those in the no major complications group (all, *p* < 0.05). Major complications were also more common when the procedure was performed by low-volume surgeons (*p* = 0.003). In multivariate logistic regression models, CCI ≥ 5 (*p* = 0.006) and low-volume surgeon (*p* = 0.004) were independent predictors of major complications. According to multivariate analysis with the Cox hazards regression, male sex, age > 70 years, CCI ≥ 5, bladder cancer stage ≥ 3, lymphovascular invasion, and experiencing major complications were significant poor prognostic factors for overall survival (all, *p* < 0.05). Conclusions: Accurate reporting of complications is necessary for preoperative counseling, identifying modifiable risk factors, and planning risk mitigation strategies. High comorbidity and low-volume surgeons were interrelated as notable risk factors for major complications. In addition to tumor-related factors, male sex, older age, and major complications significantly influence overall survival.

## 1. Introduction

Chronic kidney disease (CKD) is a substantial public health concern worldwide, with CKD stage 3–5 estimated to affect 11–13% of the general population [1]. Taiwan has the highest prevalence (3679 per million population in 2019) and the second-highest incidence (529 new cases per million population in 2019) of treated end-stage renal disease (ESRD) in the world [2]. ESRD patients on dialysis have an elevated incidence of certain cancers and death compared with the general population [3]. In Taiwan, urothelial carcinoma (UC) of the bladder is the most common malignancy in patients receiving dialysis [4].

Dialysis patients with bladder UC are more likely to have synchronous or metachronous cancer foci throughout the urinary tract and often present with more adverse pathologic features than non-dialysis patients. Radical cystectomy is the standard of care for localized muscle-invasive bladder cancer (MIBC), as well as for selected patients with high-risk non-MIBC (NMIBC), regardless of ESRD status [5]. Despite improvements in anesthetic competency, surgical techniques, and postoperative care that have reduced procedure-related complication rates, dialysis patients undergoing radical cystectomy are at a higher risk of perioperative morbidity and mortality than non-dialysis patients [6]. Sato et al. reported that the major complication rate (Clavien grades 3–5) after radical cystectomy in dialysis patients was 30%, with a mortality rate of 20% [7]. These rates are significantly higher than the 13% major complication rate and 1.5% mortality rate reported by Shabsigh et al. for the general population undergoing radical cystectomy [8]. Increased morbidity and mortality in dialysis patients may relate to a high prevalence of heart disease [9], perioperative fluid and electrolyte disturbances [10], bleeding diathesis [11], and hemodynamic instability. Although these entities are plausible explanations for the increased risk of complications, published data on predictors of outcome in ESRD patients undergoing radical cystectomy are scant.

In this study, we identify the incidence, type, and severity of perioperative complications in dialysis patients undergoing radical cystectomy for UC using a standardized complication reporting system. Our intention is to provide information to guide perioperative management and prognostication for ESRD patients contemplating radical cystectomy. Secondary endpoints include survival analysis for ESRD patients undergoing radical cystectomy. We hypothesized that known risk factors for major perioperative complications from radical cystectomy would be applicable to the ESRD population.

## 2. Methods

### 2.1. Study Population

Institutional review board (IRB) approval was obtained (IRB No. 202100779B0), and the requirement for informed consent was waived. In total, 141 patients with ESRD and comorbid bladder cancer who underwent radical cystectomy with a curative intent between January 2004 and December 2015 were identified from the medical record systems of two major cancer centers. At our institutions, radical cystectomy is recommended for dialysis patients with localized MIBC or for selected dialysis patients with recurrence or incomplete transurethral resection of NMIBC. Abdominopelvic computed tomography (CT) or magnetic resonance urography (MRU) and transurethral resection were used for preoperative staging, whereas cytology, chest CT, and bone scan were performed as indicated in select patients.

### 2.2. Pathological Evaluation

The final pathological features collected at the time of radical cystectomy were as follows: tumor stage, tumor grade, lymphovascular invasion (LVI, yes/no), tumor margin status (positive/negative), and presence of carcinoma in situ (CIS, yes/no). The tumor stage was determined according to the 8th edition of the American Joint Committee on Cancer TNM Staging System. For urothelial histologies, low and high grades were used to match the current World Health Organization/International Society of Urological Pathology’s recommended grading system. All specimens were evaluated by urological pathologists at our institutions.

### 2.3. Outcome Measures

Recommended follow-up for all patients included annual and “for cause” cross-sectional imaging (abdominopelvic CT or MRU) and chest radiography. Chest CT and bone scan were performed as indicated in select patients with advanced stage or bone pain.

The following patient, surgical, and postoperative data were collected: sex, age at surgery, body mass index (BMI), Charlson comorbidity index (CCI), prior abdominal surgery, renal replacement therapy method(s) and duration, preoperative hemoglobin, preoperative plasma potassium, year of cystectomy, American Society of Anesthesiologists (ASA) score, operative surgeon volume, operative method, operative time, intraoperative blood loss, hospitalization duration, and final pathological findings. Surgeon volume was assessed as a binary with low-volume surgeons classified as those performing < 9 cases during the study and high-volume surgeons being those with ≥ 9 cases during that interval [12].

The Clavien–Dindo classification system was used to grade surgical complications [13]. Clavien–Dindo is a standardized complication reporting system recommended by the International Consultation on Urological Diseases-European Association of Urology International Consultation on Bladder Cancer 2012 [14]. Complications were censored within the first 90 days after cystectomy or during hospitalization, whichever was longer. Major complications were classified as Clavien grades 3–5 [15]. The impact of clinical characteristics on perioperative complications was evaluated by stratifying the patients into the no major complications group (Clavien grade 0–2 complications only) and the major complications group (any Clavien grade 3–5 complications).

Survival time was calculated from the date of radical cystectomy to the time of the last follow-up or death (cancer-specific or any cause). Recurrence time was estimated from the date of radical cystectomy to the time of first recurrence (if any). Metachronous upper tract UC was not included in the recurrence-free survival analysis.

### 2.4. Statistical Analysis

All statistical comparisons were performed using SPSS version 20 (IBM Corporation, Armonk, NY, USA) or Prism version 9 (GraphPad Software, San Diego, CA, USA). In descriptive analyses, continuous and categorical variables were reported as median values with ranges and proportions, respectively. The two-tailed *t* test was used to compare the difference in continuous variables between patients who did or did not have major complications, whereas categorical variables were analyzed using Fisher’s exact test along with the chi-squared test. Univariable and multivariable logistic regressions were used to evaluate the odds ratio (OR) with a 95% confidence interval (CI) and identify factors independently associated with experiencing major complications. We used the Kaplan–Meier method with the log-rank test to analyze overall survival, cancer-specific survival, and recurrence-free survival rates after radical cystectomy. Univariate and multivariate analyses with Cox hazards regression were used to analyze the value of prognostic factors for overall, cancer-specific, and recurrence-free survival [16,17]. *p* < 0.05 was set as the threshold for statistical significance for all analyses. Because of sample size considerations, only those factors identified with *p* < 0.05 in univariable analysis were further evaluated in multivariable analysis.

## 3. Results

### 3.1. Study Population

In total, 141 patients with ESRD who received radical cystectomy during the time interval of the study were identified (Table 1). The median patient age was 64 years (range, 24–86 years).

From the study cohort, 99 (70.2%) and 42 (29.8%) patients had Clavien grade 0–2 complications (no major complications) and Clavien grade 3–5 complications (major complications), respectively. Female patients (59.6%) were slightly predominant in both groups. Patients in the major complications group were older (70 vs. 63 years, *p* = 0.010), had a higher CCI (5 vs. 4, *p* = 0.003), and had a longer hospitalization duration (14 vs. 9 days, *p* < 0.001) than patients in the no major complications group. The procedure was performed by 27 surgeons, and the median surgeon volume was 3 cases (range, 1–27 cases) during the time interval of the study. Major complication rates were higher when the procedure was performed by low-volume surgeons (<9 cases; *p* = 0.003). None of the other variables analyzed showed a statistically significant difference between groups (all, *p* > 0.05).

### 3.2. Pathological Features

In the study population, one (0.7%) patient had small cell neuroendocrine carcinoma, and two (1.4%) patients had adenocarcinoma, with the remainder having UC. A substantial minority of patients (52, 36.9%) had no residual tumor at radical cystectomy; all of these had a history of bladder cancer previously treated by transurethral resection (median, two procedures; range, one to nine procedures, Table 2). The pathological stage of the radical cystectomy specimen was MIBC in 26 (18.4%) patients, suggesting that most patients underwent the procedure for recurrent disease or incomplete transurethral resection of NMIBC. Among the 57 male patients, prostate cancer was diagnosed in 12 (21.1%) patients at radical cystoprostatectomy.

No statistically significant difference in tumor stage, tumor grade, LVI, positive surgical margin, and CIS was noted between patients who did or did not have major complications (all, *p* > 0.05).

### 3.3. Postoperative Complications

Overall, 116 (82.3%) patients experienced one or more complications. Of them, 42 (29.8%) patients had major complications (Table 3). Arteriovenous shunt dysfunction requiring surgical intervention was the most common major complication, affecting 10 (7.1%) patients. No intraoperative death was noted. Seven (7, 5.0%) patients died in the postoperative recovery period. The causes of death were intra-abdominal abscess (n = 3), cardiac arrest (n = 2), acute respiratory distress syndrome (n = 1), and pancreatic injury (n = 1). Median time to death for the seven patients was 8 days after surgery (range, 2–145 days).

### 3.4. Predictive Probability of Major Complications

Categorically coded age > 70 years (OR, 2.77, 95% CI, 1.17–6.56; *p* = 0.020), CCI ≥ 5 (OR, 3.54, 95% CI, 1.57–7.98; *p* = 0.002), surgeon volume < 9 cases (OR, 3.29, 95% CI, 1.55–6.99; *p* = 0.002), and LVI (OR, 3.96, 95% CI, 1.06–14.8; *p* = 0.041) were independent predictors of postoperative major complications in univariate logistic regression analyses (Table 4). According to the results of multivariate analyses, only CCI ≥ 5 (OR, 3.43, 95% CI, 1.43–8.23; *p* = 0.006) and low-volume surgeon (OR, 3.29, 95% CI, 1.46–7.40; *p* = 0.004) were independently associated with major complications. No variable was significantly predictive of perioperative mortality after multivariable adjustment (data not shown).

### 3.5. Recurrence and Survival

The median follow-up period after radical cystectomy was 50.7 months (range, 2 days to 179.6 months, Table 5). At the end of follow-up, 7 (5.0%) patients died of perioperative complications, 7 (5.0%) died of bladder cancer-associated causes, and 24 (17.0%) died of other causes. Bladder cancer recurrence was detected in 15 (10.6%) patients. The 5-year overall, cancer-specific, and recurrence-free survival rates were 76.0%, 93.0%, and 86.8%, respectively.

Categorically coded male sex (HR 2.36, 95% CI, 1.15–4.83; *p* = 0.019), age > 70 years (HR 2.91, 95% CI, 1.29–6.60; *p* = 0.010), CCI ≥ 5 (HR 4.03, 95% CI, 1.54–10.5; *p* = 0.004), bladder cancer stage ≥ 3 (HR 37.2, 95% CI, 6.44–215; *p* < 0.001), LVI (HR 0.1, 95% CI, 0.01–0.76; *p* = 0.026), and Clavien grade 3–5 complications (HR 3.58, 95% CI, 1.61–7.95; *p* = 0.002) were significant negative prognostic factors for overall survival in the multivariable Cox regression models. In addition, bladder cancer stage ≥ 3 (HR 6.61, 95% CI, 1.04–42.1; *p* = 0.046) and kidney transplantation (HR 6.75, 95% CI 1.09–41.7; *p* = 0.040) in multivariable analysis were the only significant unfavorable prognostic factors for cancer-specific and recurrence-free survival, respectively.

## 4. Discussion

Although many risk factors have been described and validated to help predict radical cystectomy-associated complications in the general population, information regarding perioperative complications and survival in dialysis patients with UC undergoing radical cystectomy is less and limited to small case series [7,18,19]. In the largest study using administrative data from the United States Renal Data System database, Johnson et al. identified 985 hemodialysis patients receiving radical cystectomy from different centers [20]. In these patients, surgical morbidity and mortality were significant, and 5-year overall survival was low. Notably, in that study, staging information and specific operative details were unavailable, and the complication was not reported using a standardized reporting methodology. Although our current study is not a multi-institutional collaborative study, patient care, including surgery and postoperative care, was uniform at our institutions. Furthermore, surgical complications were strictly reported according to the standardized reporting system, and survival rates were accurately recorded.

The ASA score, introduced in 1941 and originally designed to standardize patients’ physical status categories for statistical studies and for hospital records, is most frequently reported as a general surgical risk score [21]. The ASA score is a useful classification system but is limited by interobserver variability; a cohort of anesthesiologists may ascribe different ASA scores to the same patient [22]. In contrast to the ASA score, the CCI stratifies comorbidity into different risk groups by more precisely assigning scores to various illnesses, which consists of 19 clinically critical conditions with each given 1–6 points based on a low or high morbidity grade [23]. Although both CCI and ASA scores are common indicators of the methodology used to assess patient risk stratification, in our cohort, only CCI was significantly beneficial in predicting perioperative major complications after radical cystectomy rather than ASA score. Very similar results were reported by Yuh et al.; CCI (OR, 1.39, 95% CI, 1.10–1.76; *p* = 0.007), preoperative hematocrit (OR, 2.62, 95% CI, 1.19–5.78; *p* = 0.02), and neobladder diversion (OR, 3.01, 95% CI, 1.21–7.52; *p* = 0.02) in multivariable analysis were the only significant predictors of major complications [24]. Many studies have reported a correlation between major complications and the ASA score [25,26,27]. We acknowledge that an association between ASA and morbidity may not have been apparent in our data since 92.2% patients in our study had an ASA score of 3.

Existing studies have documented the effects of hospital volume on cystectomy outcomes in the general population; however, limited and conflicting data are available regarding surgeon volume [5,28]. To the best of our knowledge, the impact of surgeon volume on the prediction of complications in dialysis patients undergoing radical cystectomy has not been corroborated. In our multivariate logistic regression model, in-hospital major complications were less frequent when the procedure was performed by a high-volume surgeon (≥9 cases during the study) than by a low-volume surgeon (<9 cases during the study). This finding adds further credence to the notion that radical cystectomy candidates are best served by referrals to high-volume surgeons working in specialized centers.

The 5-year cancer-specific survival rate in our series was 93.0%, slightly higher than that in other contemporary studies (29.5–80.3%) [20,29]. The reasons for enhanced cancer-specific survival in these patients are unclear, but several potential explanations are available. First, the single-payer national health insurance system in Taiwan provides universal health coverage to >99% of Taiwan’s population and covers 92.6% of all hospitals, clinics, and other healthcare facilities [30]. Patients in Taiwan are not restricted in terms of their healthcare access. Second, due to the high incidence of upper or lower urinary tract UC in dialysis patients in Taiwan [31], these patients are under the strict medical surveillance of healthcare facilities and have frequent contact with their clinicians, especially when they develop signs concerning recurrent disease. In addition, dialysis patients tend to have pathologically more advanced bladder cancers, often necessitating radical cystectomy and exhibiting a poor prognosis, as reported in other collaborative reports [7,19]. However, in our current study, most patients receiving radical cystectomy had NMIBC (81.6%), implying a less aggressive variant.

Patients with ESRD have an elevated risk of site-specific cancer incidence and cancer death compared with people without ESRD, but over half of the known deaths were related to cardiovascular diseases [32]. In patients with ESRD receiving hemodialysis, the percentage of deaths attributable to cancer appears to be relatively low, accounting for 2.9% of deaths among patients with a known cause from the United States Renal Data System 2021 Annual Data Report [2]. Similarly, in this study, only 7 (5.0%) patients died of bladder cancer-associated causes compared with 24 (17.0%) patients who died of all other causes. Many risk factors unrelated to the dialysis procedure are associated with decreased survival among dialysis patients, including age, race, country, smoking, comorbidities, nutritional status, laboratory test, underlying cause of ESRD, performance status, psychosocial factors, salt intake, residual renal function, pre-dialysis care, and compliance [33]. Our multivariate model showed that age > 70 years and CCI ≥ 5 are significant unfavorable prognostic factors for decreased overall survival. Similarly, in Noh et al.’s study, age ≥ 70.5 years and modified CCI > 4 were the best predictors of mortality in dialysis patients, and survival HR was predicted as 4.61 compared with the overall study population [34].

Unlike other predictive factors, the effects of sex on all-cause mortality in dialysis patients have rarely been discussed. Although the reasons for the higher death rates in male patients undergoing dialysis are incompletely understood, life expectancy at birth in Taiwan was generally lower for men (76.7 years, 95% uncertainty intervals 75.0–78.5) than for women (82.8 years, 95% uncertainty intervals 81.4–84.4) in 2016 [35]. The findings are similar to those of the reports of the National Health Insurance Research Database, according to which the mortality rate of ESRD patients in Taiwan is slightly higher in men than in women at 11.1 and 10.2 deaths per hundred patient-years, respectively [36,37]. Nevertheless, the relationships between mortality and sex in dialysis patients require further investigation.

Although kidney transplantation is considered the treatment of choice in dialysis patients with UC who are cancer-free for more than 2 years after surgery, kidney transplantation recipients are known to have a higher cancer recurrence rate than the general population [38]. In our multivariate analysis, kidney transplantation was an independent predictor of subsequent cancer recurrence. Multiple factors, including genetic background, environmental factors, underlying cause of ESRD, and immunosuppressive status, may contribute to the recurrence of malignancy. Moreover, 13 of 141 (9.2%) patients had bladder cancer stage 3 or 4 compared with 2 of 4 (50%) patients receiving kidney transplantation in the current study. These data suggest that renal transplant recipients had a higher rate of being diagnosed with more advanced bladder cancer. Therefore, we recommend using the lowest possible dose of immunosuppressive agents and more rigorous medical surveillance in kidney transplant recipients developing cancers.

This is a relatively large study focusing on complications and outcomes classified according to a standardized system in a clinically challenging patient population. We acknowledge several limitations. First, this is a retrospective, nonrandomized controlled study. Selection bias is inevitable, as radical cystectomy in patients with NMIBC was chosen depending on patient preference after discussion with the treating urologist and anesthesiologist. Second, a control group is lacking. To determine the predictors of major complications and potential prognostic factors for survival, categorization of variables may result in overfitting of predictive and prognostic models. Additionally, the number of patients is too small to draw definite conclusions, especially in the major complications group. Prospective, large-scale trials are required to best determine how to optimize care for ESRD patients with UC.

## 5. Conclusions

Accurate reporting of complications is essential for preoperative counseling, identifying modifiable risk factors to reduce complication rates, and planning risk mitigation strategies. A high comorbidity index is associated with an increased risk of postoperative complications and reduces overall survival. Perioperative major complications may be minimized by directing patients to surgeons who frequently perform radical cystectomy. In addition to tumor-related factors, male sex, older age, and perioperative major complications have an impact on overall survival after radical cystectomy in dialysis patients with bladder cancer.

## Figures and Tables

**Table 1:** Clinical characteristics of dialysis patients undergoing radical cystectomy.

	Total (*n* = 141)	No Major Complication (Clavien 0–2, *n* = 99)	Major Complication (Clavien 3–5, *n* = 42)	*p* Value
Gender				0.348
Female	84 (59.6)	56 (56.6)	28 (66.7)	
Male	57 (40.4)	43 (43.4)	14 (33.3)	
Age, years, median (range)	64 (24–86)	63 (24–86)	70 (50–83)	**0.010** 0.062
<60	56 (39.7)	45 (45.5)	11 (26.2)	
60–70	33 (23.4)	23 (23.2)	10 (23.8)	
>70	52 (36.9)	31 (31.3)	21 (50.0)	
BMI, kg/m^2^, median (range)	23.0 (16.2–38.1)	22.9 (17.5–38.1)	23.3 (16.2–32.7)	0.799 0.684
<25.0	98 (69.5)	70 (70.7)	28 (66.7)	
≥25.0	40 (28.4)	27 (27.3)	13 (31.0)	
Unknown	3 (2.1)	2 (2.0)	1 (2.4)	
CCI, median (range) ^a^	5 (3–9)	4 (3–9)	5 (4–9)	**0.003** **0.002**
≤4	62 (44.0)	52 (52.5)	10 (23.8)	
≥5	79 (56.0)	47 (47.5)	32 (76.2)	
Previous abdominal surgery	73 (51.8)	52 (52.5)	21 (50.0)	0.855
Renal replacement therapy				0.984
Hemodialysis	119 (84.4)	84 (84.8)	35 (83.3)	
Peritoneal dialysis	15 (10.6)	10 (10.1)	5 (11.9)	
Kidney transplantation	4 (2.8)	3 (3.0)	1 (2.4)	
None	3 (2.1)	2 (2.0)	1 (2.4)	
Dialysis duration, years				0.646
<5	67 (47.5)	45 (45.5)	22 (52.4)	
5–10	41 (29.1)	31 (31.3)	10 (23.8)	
>10	33 (23.4)	23 (23.2)	10 (23.8)	
Hemoglobin, g/dL, median (range) ^a^	10.1 (6.0–15.7)	10.1 (6.8–15.7)	10.2 (6.0–12.7)	0.540 1.000
≤10.1	72 (51.1)	51 (51.5)	21 (50.0)	
>10.1	69 (48.9)	48 (48.5)	21 (50.0)	
Potassium, mEq/L, median (range) ^b^	4.2 (2.7–6.8)	4.2 (2.7–6.8)	4.3 (3.3–5.7)	0.815 0.090
<3.6	13 (9.2)	12 (12.1)	1 (2.4)	
3.6–5.0	86 (61.0)	56 (56.6)	30 (71.4)	
>5.0	19 (13.5)	15 (15.2)	4 (9.5)	
Unknown	23 (16.3)	16 (16.2)	7 (16.7)	
Cystectomy year				0.547
2004–2007	47 (33.3)	34 (34.3)	13 (31.0)	
2008–2011	43 (30.5)	32 (32.3)	11 (26.2)	
2012–2015	51 (36.2)	33 (33.3)	18 (42.9)	
ASA score, median (range)	3 (1–4)	3 (1–3)	3 (2–4)	0.235 0.387
1	1 (0.7)	1 (1.0)	0 (0)	
2	9 (6.4)	7 (7.1)	2 (4.8)	
3	130 (92.2)	91 (91.9)	39 (92.9)	
4	1 (0.7)	0 (0)	1 (2.4)	
Surgeon volume, median (range)	3 (1–27)			**0.003**
Low, <9 cases	62 (44.0)	35 (35.4)	27 (64.3)	
High, ≥9 cases	79 (56.0)	64 (64.6)	15 (35.7)	
Operative methods				0.839
Open	101 (71.6)	70 (70.7)	31 (73.8)	
Laparoscopic	40 (28.4)	29 (29.3)	11 (26.2)	
Operative time, min, median (range) ^a^	343 (68–676)	337 (68–660)	351 (213–676)	0.280 0.715
≤343	71 (50.4)	51 (51.5)	20 (47.6)	
>343	70 (49.6)	48 (48.5)	22 (52.4)	
Blood loss, mL, median (range) ^a^	600 (50–8600)	600 (50–6000)	600 (100–8600)	0.237 1.000
<600	68 (48.2)	48 (48.5)	20 (47.6)	
≥600	72 (51.1)	50 (50.5)	22 (52.4)	
Unknown	1 (0.7)	1 (1.0)	0 (0)	
Hospitalization, day, median (range)	9 (2–145)	9 (4–52)	14 (2–145)	**<0.001**

Data are expressed as n (%) unless otherwise specified. Significant *p*-values are shown in bold. ^a^ The cutoff values of the variables were determined according to median values in the study cohort. ^b^ Reference range of plasma potassium was 3.6–5.0 mEq/L. BMI: body mass index; CCI: Charlson comorbidity index; ASA: American Society of Anesthesiologists.

**Table 2:** Pathological features of dialysis patients undergoing radical cystectomy.

	Total (n = 141)	No Major Complication (Clavien 0–2, n = 99)	Major Complication (Clavien 3–5, n = 42)	*p* Value
Pathological tumor stage				0.372
0a/0is	26 (18.4)	18 (18.2)	8 (19.0)	
I	37 (26.2)	27 (27.3)	10 (23.8)	
II	13 (9.2)	8 (8.1)	5 (11.9)	
III	9 (6.4)	4 (4.0)	5 (11.9)	
IV	4 (2.8)	2 (2.0)	2 (4.8)	
No residual tumor	52 (36.9)	40 (40.4)	12 (28.6)	
Tumor grade ^a^				1.000
Low	5 (5.6)	3 (5.1)	2 (6.7)	
High	84 (94.4)	56 (94.9)	28 (93.3)	
Lymphovascular invasion				0.065
Absent	131 (92.9)	95 (96.0)	36 (85.7)	
Present	10 (7.1)	4 (4.0)	6 (14.3)	
Surgical margin				1.000
Negative	136 (96.5)	95 (96.0)	41 (97.6)	
Positive	5 (3.5)	4 (4.0)	1 (2.4)	
Concomitant carcinoma in situ				0.755
Absent	128 (90.8)	89 (89.9)	39 (92.9)	
Present	13 (9.2)	10 (10.1)	3 (7.1)	

Data are expressed as *n* (%). The pathologic features were determined according to the time of radical cystectomy. ^a^ Fifty-two patients with no residual tumor in the radical cystectomy procedure were excluded.

**Table 3:** Complication grade and type after radical cystectomy (n = 141).

Grade 0	25 (17.7)
Grade 1	25 (17.7)
Grade 2	49 (34.8)
Pharmacological treatment other than grade 1	25 (17.7)
Blood transfusion	18 (12.8)
Total parenteral nutrition	6 (4.3)
Grade 3	29 (20.6)
Surgical intervention	24 (17.0)
arteriovenous shunt dysfunction	10 (7.1)
rectovaginal or vaginal fistula	3 (2.1)
infection	3 (2.1)
wound dehiscence	2 (1.4)
spleen laceration or splenectomy	2 (1.4)
bowel perforation	2 (1.4)
vaginal bleeding	1 (0.7)
ischemic leg	1 (0.7)
Radiological intervention	4 (2.8)
Endoscopic intervention	1 (0.7)
Grade 4	6 (4.3)
Life-threatening single organ dysfunction	5 (3.5)
Life-threatening multiorgan dysfunction	1 (0.7)
Grade 5, death	7 (5.0)

Data are expressed as *n* (%). Complications were graded according to the Clavien classification of surgical complications.

**Table 4:** Univariate and multivariate analysis for predictive factors of postoperative major complications.

	Univariate		Multivariate	
	OR (95% CI)	*p* Value	OR (95% CI)	*p* Value
Male gender (referent: female)	0.65 (0.31–1.39)	0.265		
Age (referent: < 60 years)		0.068		0.213
60–70	1.78 (0.66–4.80)	0.256	1.72 (0.58–5.12)	0.332
>70	2.77 (1.17–6.56)	**0.020**	2.31 (0.91–5.90)	0.079
BMI ≥ 25 kg/m^2^ (referent: <25 kg/m^2^)	1.20 (0.54–2.66)	0.647		
CCI ≥ 5 (referent: ≤4) ^a^	3.54 (1.57–7.98)	**0.002**	3.43 (1.43–8.23)	**0.006**
Previous abdominal surgery (referent: absent)	0.90 (0.44–1.86)	0.784		
RRT (referent: hemodialysis)		0.932		
Kidney transplantation	0.80 (0.08–7.96)	0.849		
Peritoneal dialysis	1.20 (0.38–3.77)	0.755		
Dialysis duration (referent: <5 years)		0.648		
5–10 years	0.66 (0.28–1.59)	0.352		
>10 years	0.88 (0.36–2.19)	0.799		
Hemoglobin ≤ 10.1 g/dL (referent: >10.1 g/dL) ^a^	0.94 (0.46–1.94)	0.869		
Potassium (referent: 3.6–5.0 mEq/L) ^b^		0.130		
<3.6	0.16 (0.02–1.26)	0.081		
>5.0	0.50 (0.15–1.63)	0.250		
Cystectomy year (referent: 2004–2007)		0.549		
2008–2011	0.90 (0.35–2.29)	0.824		
2012–2015	1.43 (0.60–3.37)	0.418		
ASA score ≥ 3 (referent: ASA ≤ 2)	1.76 (0.36–8.65)	0.488		
Low-volume surgeon (referent: ≥ 9 cases) ^c^	3.29 (1.55–6.99)	**0.002**	3.29 (1.46–7.40)	**0.004**
Open method (referent: laparoscopy)	1.17 (0.52–2.63)	0.709		
Operative time > 343 min (referent: ≤ 343 min) ^a^	1.17 (0.57–2.41)	0.672		
Blood loss ≥ 600 mL (referent: <600 mL) ^a^	1.06 (0.51–2.18)	0.883		
Stage 3/4 (referent: stage ≤ 2)	3.10 (0.97–9.87)	0.055		
Tumor grade (referent: low grade)	0.75 (0.12–4.75)	0.760		
Lymphovascular invasion (referent: absent)	3.96 (1.06–14.8)	**0.041**	3.46 (0.81–14.8)	0.094
Surgical margin (referent: negative)	0.58 (0.06–5.34)	0.630		
CIS (referent: absent)	0.69 (0.18–2.63)	0.581		

Significant *p*-values are shown in bold. ^a^ The cutoff values of the variables were determined according to median values in the study cohort. ^b^ Reference range of plasma potassium was 3.6–5.0 mEq/L. ^c^ Low-volume surgeons classified as those performing fewer than 9 cases during the study. OR: odds ratio; CI: confidence interval; BMI: body mass index; CCI: Charlson comorbidity index; RRT: renal replacement therapy; ASA: American Society of Anesthesiologists; CIS: concomitant carcinoma in situ.

**Table 5:** Univariate and multivariate analysis of potential prognostic factors for overall, cancer-specific, and recurrence-free survival in dialysis patients undergoing radical cystectomy.

	Overall Survival				Cancer-Specific Survival				Recurrence-Free Survival			
	Univariate		Multivariate		Univariate		Multivariate		Univariate		Multivariate	
	HR (95% CI)	*p* Value	HR (95% CI)	*p* Value	HR (95% CI)	*p* Value	HR (95% CI)	*p* Value	HR (95% CI)	*p* Value	HR (95% CI)	*p* Value
Male gender (referent: female)	2.09 (1.10–3.95)	**0.024**	2.36 (1.15–4.83)	**0.019**	2.49 (0.56–11.1)	0.233			1.95 (0.71–5.38)	0.198		
Age (referent: <60 years)		**<0.001**		**0.023**		0.965				0.299		
60–70 years	1.60 (0.57–4.46)	0.372	1.27 (0.44–3.65)	0.659	0 (0–1000)	0.971			0.26 (0.03–2.13)	0.210		
>70 years	4.48 (2.06–9.74)	**<0.001**	2.91 (1.29–6.60)	**0.010**	1.22 (0.27–5.50)	0.792			1.36 (0.48–3.90)	0.564		
BMI ≥ 25 kg/m^2^ (referent: <25 kg/m^2^)	0.68 (0.31–1.49)	0.338			0.40 (0.05–3.32)	0.396			0.83 (0.26–2.60)	0.744		
CCI ≥ 5 (referent: ≤4) ^a^	4.30 (1.89–9.79)	**0.001**	4.03 (1.54–10.5)	**0.004**	2.26 (0.44–11.7)	0.329			2.35 (0.75–7.39)	0.143		
Previous abdominal surgery (referent: absent)	0.80 (0.42–1.51)	0.484			2.23 (0.43–11.5)	0.339			1.29 (0.46–3.62)	0.633		
RRT (referent: hemodialysis)		0.438				0.214				**0.039**		0.102
Kidney transplantation	2.10 (0.50–8.79)	0.311			6.80 (0.79–58.3)	0.080			7.19 (1.57–32.8)	**0.011**	6.75 (1.09–41.7)	**0.040**
Peritoneal dialysis	0.65 (0.20–2.12)	0.473			1.64 (0.19–14.1)	0.650			1.49 (0.33–6.80)	0.609	0.97 (0.16–5.97)	0.972
Dialysis duration (referent: <5 years)		0.356				0.713				0.597		
5–10 years	0.72 (0.34–1.52)	0.385			1.39 (0.28–6.90)	0.685			0.93 (0.31–2.85)	0.902		
>10 years	0.56 (0.24–1.31)	0.179			0.55 (0.06–5.25)	0.599			0.45 (0.10–2.13)	0.316		
Hemoglobin ≤ 10.1 g/dL (referent: >10.1 g/dL) ^a^	0.86 (0.45–1.63)	0.638			1.33 (0.30–5.94)	0.709			1.49 (0.53–4.19)	0.450		
Potassium (referent: 3.6–5.0 mEq/L) ^b^		0.906				0.684				0.978		
<3.6	1.18 (0.41–3.45)	0.758				0.989				0.981		
>5.0	0.86 (0.30–2.52)	0.789			2.13 (0.39–11.6)	0.383			0.85 (0.19–3.84)	0.834		
Cystectomy year (referent: 2004–2007)		0.909				0.496				0.846		
2008–2011	1.12 (0.53–2.38)	0.765			3.56 (0.37–34.2)	0.272			0.89 (0.24–3.32)	0.862		
2012–2015	1.20 (0.51–2.81)	0.676			3.64 (0.38–35.1)	0.265			1.27 (0.39–4.21)	0.693		
ASA score ≥ 3 (referent: ASA ≤ 2)	0.68 (0.27–1.76)	0.431			0.23 (0.04–1.18)	0.077			0.54 (0.12–2.40)	0.419		
Low surgeon volume (referent: ≥9 cases) ^c^	1.96 (1.03–3.75)	**0.042**	1.26 (0.58–2.71)	0.562	1.91 (0.43–8.55)	0.397			2.22 (0.79–6.23)	0.132		
Open method (referent: laparoscopic)	1.60 (0.73–3.49)	0.237			2.66 (0.32–22.1)	0.366			1.73 (0.49–6.12)	0.399		
Operative time > 343 min (referent: ≤343 min) ^a^	0.79 (0.42–1.51)	0.480			0.15 (0.02–1.25)	0.080			1.13 (0.41–3.11)	0.817		
Blood loss ≥ 600 mL (referent: <600 mL) ^a^	1.73 (0.90–3.35)	0.103			2.44 (0.47–12.6)	0.288			2.78 (0.88–8.72)	0.080		
Stage 3/4 (referent: stage ≤ 2)	5.54 (2.37–12.9)	**<0.001**	37.2 (6.44–215)	**<0.001**	9.12 (1.73–48.0)	**0.009**	6.61 (1.04–42.1)	**0.046**	20.0 (7.00–56.9)	**<0.001**	3.78 (0.51–28.2)	0.196
Tumor grade (referent: low grade)	0.62 (0.34–1.13)	0.117			0.37 (0.04–3.07)	0.357			0.26 (0.06–1.16)	0.077		
Lymphovascular invasion (referent: absent)	4.17 (1.60–10.9)	**0.004**	0.10 (0.01–0.76)	**0.026**	4.18 (0.50–35.2)	0.188			20.6 (7.12–59.6)	**<0.001**	4.42 (0.50–38.7)	0.180
Surgical margin (referent: negative)	8.06 (2.30–28.2)	**0.001**	1.97 (0.37–10.5)	0.425	19.2 (1.85–200)	**0.013**	8.90 (0.61–128)	0.109	21.0 (6.20–71.4)	**<0.001**	4.59 (0.65–32.6)	0.128
CIS (referent: absent)	0.73 (0.22–2.38)	0.603			1.40 (0.17–11.6)	0.758			0.66 (0.09–5.02)	0.687		
Clavien grade 3–5 (referent: 0–2)	3.43 (1.80–6.53)	**<0.001**	3.58 (1.61–7.95)	**0.002**	0.44 (0.05–3.64)	0.444			1.49 (0.51–4.36)	0.469		

Significant *p*-values are shown in bold. ^a^ The cutoff values of the variables were determined according to median values in the study cohort. ^b^ Reference range of random plasma potassium was 3.6–5.0 mEq/L. ^c^ Low-volume surgeons classified as those performing fewer than 9 cases during the study. HR: hazard ratio; CI: confidence interval; BMI: body mass index; CCI: Charlson comorbidity index; RRT: renal replacement therapy; ASA: American Society of Anesthesiologists.

## Data Availability

Data availability is limited due to institutional data protection law and confidentiality of patient data.
